# Audit on PRN Prescriptions in Adult Inpatient Female Ward

**DOI:** 10.1192/bjo.2024.524

**Published:** 2024-08-01

**Authors:** Alka Adhikari, Syeda Shah, Maya Dhaliwal, Ayesha Chalaby

**Affiliations:** 1 South West Yorkshire Yorkshire Partnership NHS Foundation Trust, Huddersfield, United Kingdom; 2 South West Yorkshire Yorkshire Partnership NHS Foundation Trust, Halifax, United Kingdom

## Abstract

**Aims:**

Pro re Nata (PRN) psychotropic medication prescription and administration play a crucial role in addressing patients’ immediate needs and medical care plans in acute mental health services. However, regarding the appropriate indication, use, and documentation of PRN medication prescription, review, and administration practices should be as per NICE guidelines to ensure patient safety and care quality.

This audit will encompass an evaluation of PRN medication prescription in acute inpatient psychiatry as per NICE guidelines and a reaudit after recommendations implementation.

**Methods:**

We made 8 sets of questionnaires based on The National Institute for Health and Care Excellence (NICE) guidelines recommendation for PRN prescription as per local trust policy. We collected data from 28 patients in the acute inpatient mental health unit for the first cycle. Data was collected from patients' records which included medicine charts, progress notes, and MDT reviews. We analyzed data from the first cycle and implemented changes in Clinical practice. This includes including these guidelines in junior doctor induction, weekly discussion with team pharmacist, adding prompts in medication chart, and weekly review of PRN medication in ward MDT. After 2 months we collected data of 25 patients for reaudit.

**Results:**

We analyzed first-cycle data, which required improvement as per AUDIT standard compliance. We implemented recommendations before reaudit. We found there were significant improvements in some areas of concern, although this was not 100 percent audit standard. This area includes a review of PRN medication prescriptions in the last 7 days (25 percent in the first cycle, 56 percent in reaudit), grouping them if both oral and intramuscular formulations were prescribed to avoid overdose (7.2 percent, 28 percent), documentation of minimum (10.7 percent, 24 percent) and maximum dose (100 percent, 100 percent) of PRN within 24 hours, documentation of indication (100 percent, 96 percent).

**Conclusion:**

The findings of this audit and recommendations after the first cycle of audit contribute to enhancing quality of PRN medication prescription practice in acute inpatient mental health services for health care professionals. Addressing potential areas of prescription, administration, and review and providing valuable recommendations and insight for improvement of patient safety and best clinical practice.

